# Comorbid Anxiety and Depression among Pregnant and Postpartum Women: A Longitudinal Population-Based Study

**DOI:** 10.1155/2024/7802142

**Published:** 2024-03-15

**Authors:** Quan Shen, Meili Xiao, Binglu Wang, Tan He, Jinxing Zhao, Jun Lei

**Affiliations:** ^1^Department of Obstetrics and Gynecology of The Third XiangYa Hospital of Central South University, Changsha, Hunan Province, China; ^2^Xiangya School Nursing of Central South University, Changsha, Hunan Province, China; ^3^Department of Nursing, Hunan Normal University School of Medicine, Changsha, Hunan Province, China

## Abstract

**Background:**

Longitudinal studies investigating the prevalence of comorbid anxiety and depression (CAD) and its risk factors during the perinatal period are limited. The objective of this longitudinal study was to describe the prevalence and risk factors of CAD among pregnant and postpartum women in China.

**Methods:**

From the Population Health Data Archive of the National Population Health Data Center, 1,941 Chinese pregnant or postpartum women who were surveyed for both depressive and anxiety symptoms during and after pregnancy were included in the study. This population-based longitudinal study was conducted between March 2017 and March 2022. The self-rating anxiety scale and self-rating depression scale were used to assess anxiety and depression symptoms at four time points throughout the perinatal period. The generalized estimation equation model was used to identify sociodemographic, obstetric, and mental health factors for CAD.

**Results:**

The prevalence of CAD was 15.67%, 8.36%, 11.64%, and 13.24% in the first, second, and third trimesters and postpartum, respectively. A higher proportion of women reporting, compared to women with single anxiety or depression, CAD during and after pregnancy were primiparas (OR = 1.32, 95% CI 1.06-1.65), having a smoking history (OR = 1.51, 95% CI 1.05-2.18), and having dissatisfied marital relationship (OR = 1.97, 95% CI 1.28-3.06). Women conceived with assisted reproductive treatment were reported to be less likely to have CAD (OR = 0.69, 95% CI 0.55-0.86).

**Conclusions:**

These findings highlight that CAD is relatively common in pregnant and postpartum women and recommend targeted interventions for higher risk women, specifically primiparas with a history of smoking and dissatisfied marital relationships.

## 1. Introduction

Anxiety and depression are common mental health problems in pregnant and postpartum women and are frequently cooccurred [1]. Previous research has suggested that comorbid anxiety and depression (CAD) has a significant negative effect on pregnancy and neonate outcomes compared to anxiety or depression alone, including nonphysiological delivery, prematurity, low birth weight, poor infant cognitive development, and mental health problems in late childhood [2–4]. Pregnant women with multiple mental health problems may also increase the number of nonscheduled antenatal care visits, emergency health care visits [5], and even suicide and infanticide [6]. Therefore, it is essential to generate evidence on the prevalence and risk factors for CAD during pregnancy and postpartum period.

Prevalence estimates of CAD varied between countries, with 6.8% in Italy [7], 9.5% in Spain [8], and 10.04% in Ethiopia [9] during the pregnancy periods, as well as 2% in Ireland [10], 6.3% in the US [11], 13.4% in Australia [12], and 22.4% in Croatia [13] during the postpartum periods. Meanwhile, a meta-analysis involving 30 different countries reported that the overall prevalence of CAD was 9.5% during pregnancy and 8.2% after delivery and also highlighted that the data mainly come from a one-time point in pregnancy or postpartum period [14]. Longitudinal studies investigating changes in the prevalence of CAD throughout the pregnancy or postpartum period are still limited. Previous research has examined the risk factors for CAD in pregnant or postpartum women, ranging from sociodemographic- to obstetric-related factors [7, 9, 10, 15], but the factors associated with CAD have not been fully examined, such as some obstetric factors (e.g., women conceived with assisted reproductive treatment (ART)) and mental feature factors (e.g., history of anxiety or depression). Meanwhile, conflicting results are also commonly reported in some risk factors. For example, Luo et al. showed that primiparas could predict an increased probability of CAD [15], while Premji et al. identified that multiparas were associated with a higher risk of CAD [16].

Given the well-documented negative effects of anxiety and depression on pregnant women and their children, CAD is an important public health issue that warrants further attention. However, evidence on the prevalence rate of CAD mainly comes from high-income countries, and it was mainly focused on a single time point in pregnancy or postpartum and did not give a more detailed description of fluctuation in CAD during the whole pregnancy and postpartum [14]. Meanwhile, the risk factors for CAD have also not been fully examined from early pregnancy to postpartum, and some factors showed inconsistent findings. Furthermore, the prevalence and factors contributing to CAD would be different due to different cultural and socioeconomic environments in various regions [17]. Therefore, our longitudinal study is aimed at identifying the prevalence and risk factors for CAD among pregnant and postpartum women in the Chinese context.

## 2. Methods

### 2.1. Study Design

This was a population-based longitudinal study design and was reported following the Strengthening the Reporting of Observational Studies in Epidemiology (STROBE) statement [18]. It was an appropriate study design to gather data on the prevalence and risk factors of CAD in pregnant and postpartum women.

### 2.2. Data Sources

Data from this longitudinal population-based study were derived from the Population Health Data Archive (PHDA) of the National Population Health Data Center (https://www.ncmi.cn/). This center is a nonprofit institution supported by the Institute of Medical Information and the Chinese Academy of Medical Sciences, which is one of the 20 national science data centers approved by the Ministry of Science and Technology and the Ministry of Finance, China [19]. The PHDA receives scientific data from science and technology projects supported by the national budget and also collects data from other multiple sources, such as medical and health institutions, research institutions, and social individuals, which are orientated to the national big data strategy and the healthy China strategy. In our study, the data come from “Science Data of People Mental Health (SDPMH)” of PHDA, which provides sociodemographic and clinical data in detail. The SDPMH data set was conducted between March 22, 2017 and March 30, 2022 [20].

### 2.3. Study Population

Overall, a total of 1,941 pregnant women over 18 years of age who were surveyed for both depressive and anxiety symptoms and had no significant missing in anxiety and depression variables were extracted from the SDPMH data set. All participants were followed at four time points, including the first trimester, second trimester, third trimester, and postpartum. Geographically, although the survey was established in a province of southwest China, there were 73 women from the other 20 provinces of China. Meanwhile, the population mainly involved seven ethnic groups, including Han, Mongolian, Manchu, Hui, Tibetan, Zhuang, and Uyghur. These indicated that the study population is somewhat nationally representative. Considering that all data was extracted from the PHDA in this study, thus, there was no required approval from the Ethics Review Committee.

### 2.4. Data Collection

The SDPMH questionnaires capture two sections directed at risk factors (sociodemographic, obstetric, and mental health variables) and outcome data (anxiety and depression). Data were collected at four time points: in the first trimester (1-13 gestational weeks), in the second trimester (14-26 gestational weeks), in the third trimester (27-40 gestational weeks), and at postpartum (after delivery).

### 2.5. Baseline Questionnaire

The baseline questionnaire collected information including sociodemographic variables (maternal age, ethnicity, occupation, residence, educational level, annual household income, marital status, negative life events, smoking history, alcohol history, marital relationships, and in-law relationships), obstetric variables (parity, gravidity, ART pregnancy, planned or unplanned pregnancy, history of cesarean delivery, history of preterm birth, history of spontaneous abortion, prepregnancy complications, regular prenatal visits, and sought mental health services), and mental health variables (history of anxiety and depression and family history of anxiety and depression). Moreover, two obstetric variables were also collected after birth, including the mode of delivery and types of breastfeeding.

### 2.6. Self-Rating Anxiety Scale

The Self-Rating Anxiety Scale (SAS) was used to measure the anxiety level of pregnant women [21]. The scale is a 20-item self-administered tool. The participants responded on a four-point scale from 1 (no or little time) to 4 (most or all of the time). The total scores ranged from 20 to 80, and higher scores indicated higher anxiety levels (the cut-off value is 50). The Cronbach's *α* coefficient was 0.91 in the measurement of pregnant women [22].

### 2.7. Self-Rating Depression Scale

The Self-Rating Depression Scale (SDS) was used to measure the depression level of pregnant women [23]. The scale includes 20 items, and the scores for each item range from 1 (no or little time) to 4 (most or all of the time). The SDS ranged from 20 to 80 and higher scores indicated a higher level of depression (the cut-off value is 50). The Cronbach's *α* coefficient was 0.89 in the measurement of pregnant women [24].

### 2.8. Data Analysis

Analysis was conducted using IBM SPSS 23.0 (Statistical Package Program for Social Sciences 23.0, SPSS Inc., Chicago, IL, USA), with a significance level of 0.05 (two-tailed). The study variables were presented descriptively using frequency and percentage. The Chi-square test or Fisher's exact test was employed to determine the differences in baseline variables between the CAD group and the single anxiety or depression group. The factors of CAD were examined using a generalized estimation equation (GEE) model [25]. Before performing the GEE model, a prior univariate analysis was used to detect potentially significant factors associated with CAD during pregnancy and after birth, and then factors with *p* < 0.1 in the univariate analysis were then included in the model. The AR (1) working correlation structure was used in the current study since this is a commonly used working correlation structure to analyze longitudinal data [26]. Measurements of the association were presented as odds ratio (OR) with 95% confidence interval (CI).

## 3. Results

### 3.1. Characteristics of the Participants

A total of 1,941 pregnant women were eventually included in this longitudinal study. Among the 1,941 women at baseline, 1878, 1941, and 1901 women completed the questionnaires in the second trimester, third trimester, and after birth, with the response rates of 96.75%, 100%, and 97.40%, respectively. No reasons were reported for lost-to-follow-up in the current dataset. The participants were aged between 18 and 47 years old, with a mean age of 30.92 years. More than half of the participants (53.63%) were multiparous, and most of the participants (88.05%) gave birth without ART. Almost 1% of the participants reported a history of anxiety and depression disorders. The detailed sociodemographic, obstetric, and mental health characteristics among participants are listed in Table 1.

### 3.2. Prevalence of CAD

**Figure 1:**
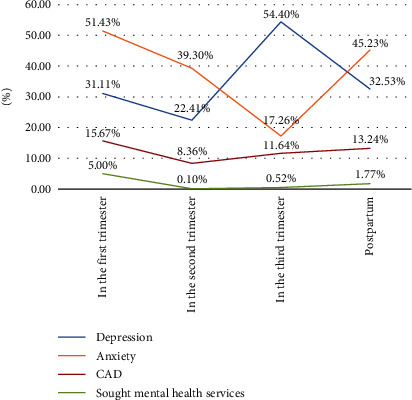
Prevalence of anxiety, depression, and comorbid of anxiety and depression.

Anxiety alone was reported by 51.43%, 39.30%, 17.26%, and 45.23% of women in the first trimester, second trimester, third trimester, and after birth. There are 31.11%, 22.41%, and 54.40% of women reporting depression alone during pregnancy, and 32.53% of women reporting depression after birth. Regarding the prevalence of CAD, it shows a U shape in general. The prevalence of CAD in the first trimester ranked the highest value (15.67%) and decreased to the lowest value (8.36%) in the second trimester and then appeared to continuously increase during the third trimester and after birth. Additionally, it should be noted that very few participants with CAD sought mental health services during pregnancy and after birth (see Figure 1).

### 3.3. Risk Factors of CAD

In the univariate analyses, significant differences between women with CAD and women with single morbidity were found in the following 14 variables: residence, annual household income, smoking history, alcohol history, parity, gravidity, ART delivery, history of spontaneous abortion, history of preterm birth, history of cesarean delivery, family history of anxiety, marital relationships, in-law relationships, and time points. The result of the GEE model indicated that primipara (OR = 1.32, 95% CI 1.06-1.65), women with a smoking history (OR = 1.51, 95% CI 1.05-2.18), and women with dissatisfied marital relationships (OR = 1.97, 95% CI 1.28-3.06) are more likely to develop CAD during pregnancy and after birth. The ART women (OR = 0.67, 95% CI 0.50-0.89) are less likely to develop CAD than women with spontaneous pregnancy. Compared to postpartum, women in the first trimester tend to develop CAD but without significant differences (OR = 1.10, 95% CI 0.75-1.62); however, women in the second trimester are less likely to develop CAD (OR = 0.69, 95% CI 0.55-0.86). The details are listed in Table 2.

## 4. Discussion

This longitudinal study provides new insight into changes in the prevalence of CAD throughout the perinatal period, and it also performs a comprehensive analysis of risk factors in sociodemographic, obstetric, and mental health dimensions. In our study, the prevalence of CAD showed a U shape, and it was reported to be 15.67%, 8.36%, 11.64%, and 13.24% in the first, second, and third trimesters and postpartum periods, respectively. The risk factors for CAD reported were primiparas, smoking history, and marital satisfaction relationships.

Our finding of CAD prevalence was relatively higher than a recent meta-analysis showing a rate of 11.6%, 10.6%, 9.5%, and 9.4% in the first, second, third, and postpartum periods, respectively [14]. The discrepancies might be due to differences in the anxiety and depression measurement scales and geographical settings. Previous meta-analysis mainly used the State-Trait Anxiety Inventory for anxiety and the Edinburgh Postnatal Depression Scale for depression [14], and our study used SAS and SDS to measure anxiety and depression, respectively. However, it should be noted that SAS and SDS also demonstrated good discriminant and predictive validity and are also the frequently used self-report measures for the evaluation of anxiety and depression in the field of perinatal mental health research [22, 27]. This meta-analysis mainly included studies located in high-income countries, but low- and middle-income countries (e.g., China) may present higher levels of anxiety and depression during the perinatal period, as there is limited availability of mental health services [28]. Moreover, regarding the social and cultural environment in China, the China society generally considers pregnancy as a woman's nature and responsibility solely [29], and pregnant women are required to follow the traditional pregnancy dietary and behavioral restrictions [30]. This suggests that women living in this social-cultural environment are more likely to develop psychological morbidity and may consequently present higher levels of CAD [30].

Our study also found that the prevalence of CAD presents a U shape during the perinatal period. This finding was also differed from the result of this meta-analysis which showed that the prevalence of CAD decreased between the first and third trimesters [14]. The difference may be due to different countries (mainly high-income countries vs middle-income country) and different study designs. Our study used a population-based longitudinal study design, and the meta-analysis mainly used a cross-sectional design at one-time point. Additional longitudinal research is required to better understand the changes in CAD prevalence over time in pregnant and postpartum women. However, in accordance with this meta-analysis, women in the first trimester present the highest prevalence of CAD, highlighting that early detection and intervention (e.g., mindfulness-based cognitive therapy) in the first trimester are needed in future research and clinical practice [31]. Additionally, compared to women in the postpartum period, women in the second trimester are less likely to develop CAD. This finding showed that the second trimester may be a relatively safe stage as previous research has suggested that mental health problems (e.g., anxiety and depression) occur less commonly in the second trimester (compared to the first and third trimesters) [32].

In accordance with a previous cross-sectional study [15], primiparas are more likely to develop CAD during the perinatal period. It may be because the first pregnancy is a stressful period, and primiparas probably have little childcare experience and lack self-confidence in their maternal role [33]. Our finding was also consistent with a previous study reporting that women with smoking habits have been associated with clinically significant depressive and anxiety symptoms [34], which could be explained that these women may worry more about the safety of infants since smoking history has been associated with negative impacts on maternal and infant health [35]. Meanwhile, dissatisfaction with marital relationships was a significant risk factor for CAD, and a previous review also reported that dissatisfaction with a family relationship can act as a trigger for the development of anxiety disorders [36].

Interestingly, our study revealed that ART women with single morbidity were less likely to develop CAD during the perinatal period. Previous studies have verified that mothers who successfully become pregnant with ART suffered less psychological distress than mothers with spontaneous pregnancy [37, 38] and provided explanations from women and their family perspectives. On the one hand, the course of infertility and the long wait to conceive may change the opinion of women, and they considered pregnancy as a more positive and relaxing process compared to the infertility process [39]. Meanwhile, the arrival of the much-hoped-for pregnancy may also change the lifestyles of ART women, including quitting smoking, adopting a healthy diet, and taking good care of their bodies [39]. These would be helpful in reducing the likelihood of developing anxiety and depression during pregnancy [40]. On the other hand, after getting through the infertility crisis, expectant mothers received stable marital relationships and social support from their families (e.g., partner and parents) [41], which helped increase their maternal confidence and decrease the likelihood of developing CAD [36, 42]. However, there has always been debate about evidence on the association between maternal psychological distress (e.g., anxiety and depression) and ART treatment, and there is scarce evidence on the CAD context, highlighting further investigation in future longitudinal studies.

Additionally, our findings differed from those of an earlier study that reported that women with CAD were more likely to be lower educated, lower income, not married, and of younger age [9, 10, 36]. This may be due to the fact that most of the participants in our study involved women who had higher levels of education, had a better financial situation, and are married generally, and therefore, the relationships between CAD and some sociodemographic factors are likely to be underestimated. Another consideration may be due to the current controversy about some risk factors. A cohort study indicated that young age is not itself a risk factor and that parity, not age, was associated with perinatal anxiety and depression [43]. The association between maternal age and CAD could be explored in future studies. Furthermore, the different measurement times of anxiety and depression were also a reason for the differences. In addition to focusing on a single time (e.g., third trimester) in previous studies, our study also concentrated on changes in CAD during the perinatal period and analyzed it using a GEE model.

### 4.1. Strengths and Limitation

This is the first study to investigate CAD from early pregnancy to postpartum at multitime points, which extended previous studies focusing on CAD at a single time point during perinatal periods, and thus addressing an important knowledge gap and providing a better understanding of the prevalence of CAD in the Chinese context. We also used the GEE model to analyze this longitudinal study, which is a proper analysis method for the longitudinal design. Moreover, our study performed a comprehensive analysis of sociodemographic, obstetric, and mental health variables, which makes a valuable contribution to the existing literature.

However, limitations in the current study should be acknowledged. Firstly, the participants included in the study are mainly highly educated and have higher incomes, which limits the generalizability of the findings. Another consideration is the possible role of attrition, which may also influence the internal validity of our findings. Secondly, the anxiety and depression were measured based on self-report questionnaires without supplementing that assessment with a diagnostic interview enabling DSM-5 diagnosis for anxiety and depression, and meanwhile, there are special instruments to measure perinatal depression (e.g., EPDS). However, a primary assessment of anxiety and depression using valid and feasible self-rating scales, including SAS and SDS, should be considered a valid option for research purposes [22, 44]. Thirdly, it is important to note that there may be other influential factors for understanding CAD, such as physical activity patterns [45–47], traumatic birth [48, 49], social support [42, 50], sleep quality [51, 52], and self-efficacy [53, 54]. Future research could extend existing findings by investigating the relationship between these variables and CAD in pregnant and postpartum women, and the development of intervention strategies for CAD should proactively take the aforementioned variables into consideration [55, 56]. Finally, these data were collected during the whole pregnancy period and after birth, and the long-term outcome of CAD during the postpartum period should be explored.

## 5. Conclusion

Given the negative consequences of comorbidity, there is limited research on perinatal comorbid depression and anxiety. The findings of the current longitudinal study suggest that CAD is relatively common in pregnant and postpartum women, especially for women in the first trimester. Our findings also suggest that women who are primiparas, have a smoking history, and have had dissatisfied marital relationships are at higher risk of developing CAD during the perinatal period. Thus, incorporating psychological screening and counseling as part of routine perinatal care visits, especially for high-risk women, is imperative to minimize the risk of CAD in pregnant and postpartum women.

## Figures and Tables

**Table 1:** Baseline characteristics of pregnant and postpartum women (*N* = 1,941).

Variables	*n* (%)	Variables	*n* (%)
Sociodemographic variables			
Maternal age (in years)		Occupation	
18-24	88 (4.53)	Homemaker	886 (45.65)
25-35	1463 (75.37)	Employed	377 (19.42)
≥35	390 (20.10)	Unemployed	678 (34.93)
Ethnicity		Current residence	
Han	1914 (98.61)	Urban	1507 (77.64)
Other	27 (1.39)	Rural	434 (22.36)
Education level		Annual household income (RMB)	
Primary school or below	284 (14.63)	<30,000	176 (9.07)
High school	793 (40.86)	30,000-60,000	465 (23.96)
Bachelor	754 (38.84)	60,000-100,000	682 (35.13)
Postgraduate and above	110 (5.67)	>100,000	618 (31.84)
Marital status		Negative life events	
Married	1929 (99.38)	Yes	529 (27.25)
Single/divorced	12 (0.62)	No	1412 (72.75)
Smoking history		Alcohol history	
Yes	85 (4.38)	Yes	187 (9.63)
No	1856 (95.62)	No	1754 (90.37)
Marital relationships^a^		In-law relationships⁣^∗^^b^	
Great satisfaction	790 (41.00)	Great satisfaction	572 (29.71)
Relatively satisfaction	805 (41.77)	Relatively satisfaction	866 (44.99)
Average satisfaction	213 (11.05)	Average satisfaction	393 (20.41)
Dissatisfaction	51 (2.65)	Dissatisfaction	60 (3.12)
Severe dissatisfaction	68 (3.53)	Severe dissatisfaction	34 (1.77)
Obstetric variables			
Parity		Gravidity	
Primiparity	900 (46.37)	Primigravida	761 (39.21)
Multiparity	1041 (53.63)	Multigravida	1180 (60.79)
ART pregnancy		Planned pregnancy	
Yes	232 (11.95)	Yes	1339 (68.99)
No	1709 (88.05)	No	602 (31.01)
History of cesarean		History of preterm birth	
Yes	595 (30.65)	Yes	47 (2.42)
No	1346 (69.35)	No	1894 (97.58)
History of spontaneous abortion		Prepregnancy complications	
Yes	418 (21.54)	Yes	129 (6.65)
No	1523 (78.46)	No	1812 (93.35)
Regular prenatal visits		Sought mental health services	
Yes	1006 (51.83)	Yes	149 (7.68)
No	935 (48.17)	No	1792 (92.32)
Delivery mode		Breastfeeding types	
Cesarean section	595 (30.65)	Exclusive	277 (14.27)
Vaginal delivery	1346 (69.35)	Mixed/formula	1664 (85.73)
Mental health variables			
History of anxiety		History of depression	
Yes	13 (0.67)	Yes	14 (0.72)
No	1928 (99.33)	No	1927 (99.28)
Family history of anxiety		Family history of depression	
Yes	14 (0.72)	Yes	6 (0.31)
No	1927 (99.28)	No	1935 (99.69)

Note. ART: assisted reproductive treatment. ⁣^∗^Relationship between mothers-in-law and daughters-in-law. ^a^The valid data is 1927 married women with two missing data. ^b^The valid data is 1925 married women with four missing data.

**Table 2:** Risk factors of CAD in pregnancy and after birth using the general estimating equation model.

Variables	OR [95% CI]	*p* value	Variables	OR [95% CI]	*p* value
Residence			Family history of anxiety		
Urban	1.19 [0.94, 1.50]	0.146	Yes	1.56 [0.88, 2.85]	0.129
Rural	Reference	—	No	Reference	—
Smoking history			Alcohol history		
Yes	1.51 [1.05, 2.18]	0.028⁣^∗^	Yes	1.00 [0.75, 1.33]	0.985
No	Reference	—	No	Reference	—
Annual household income			Time point		
>100,000	1.21 [0.85, 1.74]	0.292	At first trimester	1.10 [0.75, 1.62]	0.625
60,000-100,000	1.15 [0.81,1.65]	0.432	At second trimester	0.69 [0.55, 0.86]	0.001⁣^∗^
30,000-60,000	0.91 [0.62,1.32]	0.610	At third trimester	0.89 [0.73, 1.10]	0.279
<30,000	Reference	—	Postpartum	Reference	—
Parity			Gravidity		
Primiparity	1.32 [1.06, 1.65]	0.013⁣^∗^	Primigravida	0.97 [0.79, 1.18]	0.681
Multiparity	Reference	—	Multigravida	Reference	—
Assisted reproduction			Cesarean history		
Yes	0.67 [0.50, 0.89]	0.006⁣^∗^	Yes	1.00 [0.78, 1.27]	0.960
No	Reference	—	No	Reference	—
Spontaneous abortion history			Preterm birth history		
Yes	0.81 [0.64, 1.02]	0.070	Yes	1.31 [0.76, 2.25]	0.334
No	Reference	—	No	Reference	—
Marital satisfaction			In-law relationships		
Severe dissatisfaction	1.01 [0.60, 1.71]	0.972	Severe dissatisfaction	0.50 [0.24, 1.05]	0.066
Dissatisfaction	1.97 [1.28, 3.06]	0.002⁣^∗^	Dissatisfaction	0.75 [0.45, 1.24]	0.266
Average satisfaction	0.98 [0.69, 1.41]	0.928	Average satisfaction	0.78 [0.57, 1.05]	0.104
Relatively satisfaction	1.00 [0.80, 1.24]	0.988	Relatively satisfaction	0.81 [0.64, 1.02]	0.077
Great satisfaction	Reference	—	Great satisfaction	Reference	—

Note. ⁣^∗^*p* < 0.05.

## Data Availability

The datasets in the current study are available upon reasonable request by contacting the National Population Health Data Center.

## References

[1] Biaggi A., Conroy S., Pawlby S., Pariante C. M. (2016). Identifying the women at risk of antenatal anxiety and depression: a systematic review. *Journal of Affective Disorders*.

[2] Field T., Diego M., Hernandez-Reif M. (2010). Comorbid depression and anxiety effects on pregnancy and neonatal outcome. *Infant Behavior and Development*.

[3] Yang S., Yang R., Liang S. (2017). Symptoms of anxiety and depression during pregnancy and their association with low birth weight in Chinese women: a nested case control study. *Archives of Women's Mental Health*.

[4] Uguz F., Yakut E., Aydogan S., Bayman M. G., Gezginc K. (2019). The impact of maternal major depression, anxiety disorders and their comorbidities on gestational age, birth weight, preterm birth and low birth weight in newborns. *Journal of Affective Disorders*.

[5] Bitew T., Hanlon C., Kebede E., Medhin G., Fekadu A. (2016). Antenatal depressive symptoms and maternal health care utilisation: a population-based study of pregnant women in Ethiopia. *BMC Pregnancy and Childbirth*.

[6] Vichi M., Berardelli I., Pompili M. (2021). Completed suicide during pregnancy and postpartum. *Annali dell'Istituto Superiore di Sanità*.

[7] Cena L., Gigantesco A., Mirabella F. (2021). Prevalence of comorbid anxiety and depressive symptomatology in the third trimester of pregnancy: analysing its association with sociodemographic, obstetric, and mental health features. *Journal of Affective Disorders*.

[8] González-Mesa E., Kabukcuoglu K., Blasco M. (2020). Comorbid anxiety and depression (CAD) at early stages of the pregnancy. A multicultural cross-sectional study. *Journal of Affective Disorders*.

[9] Bante A., Mersha A., Zerdo Z., Wassihun B., Yeheyis T. (2021). Comorbid anxiety and depression: prevalence and associated factors among pregnant women in Arba Minch Zuria district, Gamo zone, southern Ethiopia. *PLoS One*.

[10] Hannon S., Gartland D., Higgins A. (2023). Physical health and comorbid anxiety and depression across the first year postpartum in Ireland (MAMMI study): a longitudinal population-based study. *Journal of Affective Disorders*.

[11] Farr S. L., Dietz P. M., O'Hara M. W., Burley K., Ko J. Y. (2014). Postpartum anxiety and comorbid depression in a population-based sample of women. *Journal of Women's Health*.

[12] Ramakrishna S., Cooklin A. R., Leach L. S. (2019). Comorbid anxiety and depression: a community-based study examining symptomology and correlates during the postpartum period. *Journal of Reproductive and Infant Psychology*.

[13] Nakić Radoš S., Tadinac M., Herman R. (2018). Anxiety during pregnancy and postpartum: course, predictors and comorbidity with postpartum depression. *Acta Clinica Croatica*.

[14] Falah-Hassani K., Shiri R., Dennis C. L. (2017). The prevalence of antenatal and postnatal co-morbid anxiety and depression: a meta-analysis. *Psychological Medicine*.

[15] Luo Z., Xue L., Ma L., Liu Z. (2021). Comorbid anxiety and depression and related factors among pregnant and postpartum Chinese women during the coronavirus disease 2019 pandemic. *Frontiers in Psychology*.

[16] Premji S. S., Lalani S., Shaikh K. (2020). Comorbid anxiety and depression among pregnant Pakistani women: higher rates, different vulnerability characteristics, and the role of perceived stress. *International Journal of Environmental Research and Public Health*.

[17] Waqas A., Raza N., Lodhi H. W., Muhammad Z., Jamal M., Rehman A. (2015). Psychosocial factors of antenatal anxiety and depression in Pakistan: is social support a mediator?. *PLoS One*.

[18] von Elm E., Altman D. G., Egger M., Pocock S. J., Gøtzsche P. C., Vandenbroucke J. P. (2008). The Strengthening the Reporting of Observational Studies in Epidemiology (STROBE) statement: guidelines for reporting observational studies. *Journal of Clinical Epidemiology*.

[19] National Population Health Data Center (2010). Introduction of National Population Health Data Center. https://www.ncmi.cn/phda/support.html?type=aboutus.

[20] Department of Health Science and Technology of National Health Commission (2022). *Science Data of People Mental Health; Population Health Data Archive of the National Population Health Data Center*.

[21] Zung W. W. K. (1971). A rating instrument for anxiety disorders. *Psychosomatics*.

[22] Muyiduli X., Wang S., Mo M. (2021). Changing patterns of prenatal depression and anxiety status in different trimesters and modified form of Zung scales for pregnant women. *International Journal of Psychiatry in Clinical Practice*.

[23] Zung W. W. K. (1965). A self-rating depression scale. *Archives of General Psychiatry*.

[24] Sun S., Hao Y., Qian J., Wang F., Sun Y., Yu X. (2022). Incidence and predictors of paternal anxiety and depression following fetal abnormalities requiring pregnancy termination: a cross-sectional study in China. *BMC Pregnancy and Childbirth*.

[25] Jin P. H., Chen F. (2003). *Medical statistical method*.

[26] Gosho M., Hamada C., Yoshimura I. (2011). Modifications of QIC and CIC for selecting a working correlation structure in the generalized estimating equation method. *Japanese Journal of Biometrics*.

[27] Dong H., Hu R., Lu C. (2021). Investigation on the mental health status of pregnant women in China during the pandemic of COVID-19. *Archives of Gynecology and Obstetrics*.

[28] Rathod S., Pinninti N., Irfan M. (2017). Mental health service provision in low- and middle-income countries. *Health Services Insights*.

[29] Yao H., Chan C. H. Y., Chan C. L. W. (2018). Childbearing importance: a qualitative study of women with infertility in China. *Research in Nursing & Health*.

[30] Lau Y. (2012). Traditional Chinese pregnancy restrictions, health-related quality of life and perceived stress among pregnant women in Macao, China. *Asian Nursing Research*.

[31] Zemestani M., Fazeli Nikoo Z. (2020). Effectiveness of mindfulness-based cognitive therapy for comorbid depression and anxiety in pregnancy: a randomized controlled trial. *Archives of Women's Mental Health*.

[32] Lee A. M., Lam S. K., Sze Mun Lau S. M., Chong C. S. Y., Chui H. W., Fong D. Y. T. (2007). Prevalence, course, and risk factors for antenatal anxiety and depression. *Obstetrics and Gynecology*.

[33] Ferber S. G. (2004). The nature of touch in mothers experiencing maternity blues: the contribution of parity. *Early Human Development*.

[34] Tojal C., Costa R. (2020). Anxiety and depression symptoms among pregnant women with different smoking habits. *Psychology, Health & Medicine*.

[35] Stock S. J., Bauld L. (2020). Maternal smoking and preterm birth: an unresolved health challenge. *PLoS Medicine*.

[36] Kasalova P., Prasko J., Holubova M. (2018). Anxiety disorders and marital satisfaction. *Neuro Endocrinology Letters*.

[37] Verhaak C. M., Smeenk J. M. J., Evers A. W. M., Kremer J. A. M., Kraaimaat F. W., Braat D. D. M. (2007). Women's emotional adjustment to IVF: a systematic review of 25 years of research. *Human Reproduction Update*.

[38] Yoshimasu K., Sato A., Miyauchi N. (2018). Lack of association between receiving ART treatment and parental psychological distress during pregnancy: preliminary findings of the Japan Environment and Children's Study. *Reproductive Biomedicine & Society Online*.

[39] René C., Landry I., de Montigny F. (2022). Couples’ experiences of pregnancy resulting from assisted reproductive technologies: a qualitative meta-synthesis. *International Journal of Nursing Studies Advances*.

[40] van Lee L., Chia A., Phua D. (2020). Multiple modifiable lifestyle factors and the risk of perinatal depression during pregnancy: findings from the GUSTO cohort. *Comprehensive Psychiatry*.

[41] Huang M. Z., Kao C. H., Lin K. C., Hwang J. L., Puthussery S., Gau M. L. (2019). Psychological health of women who have conceived using assisted reproductive technology in Taiwan: findings from a longitudinal study. *BMC Womens Health*.

[42] Bedaso A., Adams J., Peng W., Sibbritt D. (2021). The relationship between social support and mental health problems during pregnancy: a systematic review and meta-analysis. *Reproductive Health*.

[43] Nakamura Y., Okada T., Morikawa M. (2020). Perinatal depression and anxiety of primipara is higher than that of multipara in Japanese women. *Scientific Reports*.

[44] Zhou Y., Huang J., Baker P. N., Liao B., Yu X. (2022). The prevalence and associated factors of prenatal depression and anxiety in twin pregnancy: a cross-sectional study in Chongqing, China. *BMC Pregnancy and Childbirth*.

[45] Borodulin K. M., Evenson K. R., Wen F., Herring A. H., Benson A. M. (2008). Physical activity patterns during pregnancy. *Medicine and Science in Sports and Exercise*.

[46] Petrovic D., Perovic M., Lazovic B., Pantic I. (2016). Association between walking, dysphoric mood and anxiety in late pregnancy: a cross-sectional study. *Psychiatry Research*.

[47] Cai C., Busch S., Wang R., Sivak A., Davenport M. H. (2022). Physical activity before and during pregnancy and maternal mental health: a systematic review and meta-analysis of observational studies. *Journal of Affective Disorders*.

[48] Zaers S., Waschke M., Ehlert U. (2008). Depressive symptoms and symptoms of post-traumatic stress disorder in women after childbirth. *Journal of Psychosomatic Obstetrics and Gynaecology*.

[49] Ertan D., Hingray C., Burlacu E., Sterlé A., El-Hage W. (2021). Post-traumatic stress disorder following childbirth. *BMC Psychiatry*.

[50] Bedaso A., Adams J., Peng W., Sibbritt D. (2021). The association between social support and antenatal depressive and anxiety symptoms among Australian women. *BMC Pregnancy and Childbirth*.

[51] Fu T., Wang C., Yan J., Zeng Q., Ma C. (2023). Relationship between antenatal sleep quality and depression in perinatal women: a comprehensive meta-analysis of observational studies. *Journal of Affective Disorders*.

[52] Pascal R., Casas I., Genero M. (2023). Maternal stress, anxiety, well-being, and sleep quality in pregnant women throughout gestation. *Journal of Clinical Medicine*.

[53] Wensu Z., Xidi Z., Shaojie L. (2021). Does the presence of anxiety and depression symptoms mediate the association between family functions and self-efficacy in pregnant women in the third trimester?: a community-based cross-sectional survey. *Frontiers in Psychiatry*.

[54] Savory N. A., Hannigan B., John R. M., Sanders J., Garay S. M. (2021). Prevalence and predictors of poor mental health among pregnant women in Wales using a cross-sectional survey. *Midwifery*.

[55] He L., Soh K. L., Huang F. (2023). The impact of physical activity intervention on perinatal depression: a systematic review and meta-analysis. *Journal of Affective Disorders*.

[56] Asadzadeh L., Jafari E., Kharaghani R., Taremian F. (2020). Effectiveness of midwife-led brief counseling intervention on post-traumatic stress disorder, depression, and anxiety symptoms of women experiencing a traumatic childbirth: a randomized controlled trial. *BMC Pregnancy and Childbirth*.

